# Textpresso: An Ontology-Based Information Retrieval and Extraction System for Biological Literature

**DOI:** 10.1371/journal.pbio.0020309

**Published:** 2004-09-21

**Authors:** Hans-Michael Müller, Eimear E Kenny, Paul W Sternberg

**Affiliations:** **1**Division of Biology and Howard Hughes Medical Institute, California Institute of TechnologyPasadena, CaliforniaUnited States of America

## Abstract

We have developed Textpresso, a new text-mining system for scientific literature whose capabilities go far beyond those of a simple keyword search engine. Textpresso's two major elements are a collection of the full text of scientific articles split into individual sentences, and the implementation of categories of terms for which a database of articles and individual sentences can be searched. The categories are classes of biological concepts (e.g., gene, allele, cell or cell group, phenotype, etc.) and classes that relate two objects (e.g., association, regulation, etc.) or describe one (e.g., biological process, etc.). Together they form a catalog of types of objects and concepts called an ontology. After this ontology is populated with terms, the whole corpus of articles and abstracts is marked up to identify terms of these categories. The current ontology comprises 33 categories of terms. A search engine enables the user to search for one or a combination of these tags and/or keywords within a sentence or document, and as the ontology allows word meaning to be queried, it is possible to formulate semantic queries. Full text access increases recall of biological data types from 45% to 95%. Extraction of particular biological facts, such as gene-gene interactions, can be accelerated significantly by ontologies, with Textpresso automatically performing nearly as well as expert curators to identify sentences; in searches for two uniquely named genes and an interaction term, the ontology confers a 3-fold increase of search efficiency. Textpresso currently focuses on Caenorhabditis elegans literature, with 3,800 full text articles and 16,000 abstracts. The lexicon of the ontology contains 14,500 entries, each of which includes all versions of a specific word or phrase, and it includes all categories of the Gene Ontology database. Textpresso is a useful curation tool, as well as search engine for researchers, and can readily be extended to other organism-specific corpora of text. Textpresso can be accessed at http://www.textpresso.org or via WormBase at http://www.wormbase.org.

## Abstract 2

With the increasing availability of full-text scientific papers online, new tools, such as Textpresso, will help to extract information and knowledge from research literature

## Introduction

Text-mining tools have become indispensable for the biomedical sciences. The increasing wealth of literature in biology and medicine makes it difficult for the researcher to keep up to date with ongoing research. This problem is worsened by the fact that researchers in the biomedical sciences are turning their attention from small-scale projects involving only a few genes or proteins to large-scale projects including genome-wide analyses, making it necessary to capture extended biological networks from literature. Most information of biological discovery is stored in descriptive, full text. Distilling this information from scientific papers manually is expensive and slow, if the full text is available to the researcher at all. We therefore wanted to develop a useful text-mining tool for full-text articles that allows an individual biologist to locate efficiently information of interest.

The natural language processing field distinguishes information retrieval from information extraction. Information retrieval recovers a pertinent subset of documents. Most such retrieval systems use searches for keywords. Many Internet search engines are of this type, such as PubMed (http://www.ncbi.nlm.nih.gov/entrez/query.fcgi). Information extraction is the process of obtaining pertinent information (facts) from documents. The facts can concern any type of biological object (entity), events, or relationships among entities. Useful measures of the performance of retrieval and extraction systems are recall and precision. In the case of retrieval, recall is the number of pertinent documents returned compared to all pertinent documents in the corpus of text. Precision is the number of pertinent documents compared to the total number of documents returned. A fully attentive reader would have complete recall, but low precision, because he has to read the whole body of text to find information. The emphasis for most applications is on recall, and we thus sought a system with high recall and as high precision as possible.

Attempts to annotate gene function automatically include statistical approaches, such as cooccurrence of biological entities with a keyword or Medical Subject Heading term (Stapley and Benoit 2000; Jenssen et al. 2001). These methods have high recall and low precision, as no effort is being made to identify the kind of relationship as it occurs in the literature. Another approach has involved semantic and/or syntactic text-pattern recognition methods with a keyword representing an interaction (Sekimizu et al. 1998; Thomas et al. 2000; Friedman et al. 2001; Ono et al. 2001). They have high precision but low recall, because recognition patterns are usually too specific. Other machine learning approaches have classified abstracts and sentences for relevant interactions, but have not extracted information (Marcotte et al. 2001; Donaldson et al. 2003). For a more detailed report of these and related projects, see reviews by Andrade and Bork (2000), de Bruijn and Martin (2002), and Staab (2002).

The precision of a keyword search can be increased by searching for combinations of keywords. For example, a researcher might construct a search for “anchor cell” and the gene name “lin-12” because he is interested in learning whether *lin-12* plays a role in the anchor cell. However, there are many potential ways to describe the same concept or biological entity. Also, one often wants to search for a category of terms such as any gene or any body part. In this case, the intended search might be of a more general nature: If the researcher asks which genes are of interest in the anchor cell at all, he might have a hard time typing in all the known gene names (either one by one or concatenated with the Boolean operator “or”) in combination with the cell name. We therefore sought to develop a system that uses categories of terms such as “gene,” “cell,” or “biological process.” We established these categories of terms and organized them as an ontology, a catalog of types of objects and concepts and their relationships. The categories impart a semantic quality to searches, because the categories are based on the meaning of the entries.

In many cases literature databases only contain bibliographic information and abstracts. The latter suffer from the constraint of information compression and convolution imposed by a word limit. Access to the full text of articles is critical for sufficient coverage of facts and knowledge in the literature and for their retrieval (Blaschke and Valencia 2001); our results confirm these findings. We wanted to use the Caenorhabditis elegans literature as a test case for developing a useful information extraction system. C. elegans has a relatively small literature, so in principle we could use it to test a complete, well-defined corpus.

We also wanted to support a new database curation effort involving manual literature curation (Stein et al. 2001). Literature curation consists of identifying scientific data in literature and depositing them in an appropriate manner in a database. One extreme curation method is to read through the whole corpus of literature, identifying and extracting all significant information. This approach has the advantage that quality control of the data is done to the highest degree, based on human expertise. However, the volume and growth of biological literature makes it hard to keep the biological database up to date. In addition, data in literature may be missed by oversight, an inevitable flaw of purely human curation. The other extreme curation method is to extract data automatically. We therefore wanted a system that uses the computer to assist the curators.

Our system is defined by two key components: the introduction of an ontology and the searchability of full text. The ontology is organized into categories that facilitate broader searches of biological entities as illustrated above. To be useful, it should also contain other categories that are not composed of biological entities, but describe relationships between entities. We sought to offer the user an opportunity to query the literature in the framework of the ontology such that it returns sentences for inspection by the user. We hypothesized that searching the corpus of text with a combination of categories of an ontology could facilitate a query that contains the meaning of a question in a much better way than with keywords alone. For example, if there is a “gene” category containing all gene names and a “regulation” category that includes all terms (nouns, verbs, adjectives, etc.) describing regulation, searching for (at least) two instances of the category gene and one instance of the category regulation in a sentence increases the chance that the search engine will return a sentence describing a gene-gene regulation. The search could then be limited by using a particular gene name as a keyword to get a list of genes that regulate or are regulated by that particular gene.

## Results

We have developed a text processing system, Textpresso, that splits papers into sentences, and sentences into words or phrases. Each word or phrase is then labeled using the eXtensible Markup Language (XML) according to the lexicon of our ontology (described below). We then index all sentences with respect to labels and words to allow a rapid search for sentences that have a desired label and/or keyword. The labels fall into 33 categories that comprise the Textpresso ontology. We built a database of 3,800 C. elegans papers, bibliographic information from WormBase, abstracts of C. elegans meetings and the Worm Breeder's Gazette, and some additional links and WormBase entities. See Materials and Methods for details on the database preparation.

### Textpresso Ontology

Abstracts, titles, and full texts in the Textpresso system are processed for the purpose of marking them up semantically by the ontology we constructed. An ontology is a catalog of types of objects and (abstract) concepts devised for the purpose of discussing a domain of interest. An ontology helps to clarify a domain's semantics for everyday use, as is nicely demonstrated by Gene Ontology (GO; The Gene Ontology Consortium 2000). Although GO terms are not intended as a representation of natural language prose, they are a rich source of biologically meaningful terms and synonyms. They are the foundations for three corresponding categories in Textpresso, which are added to its 30 other categories. GO terms comprise approximately 80% of the lexicon.

The first group of categories in the Textpresso ontology consists of biological entities: It contains the categories gene, transgene, allele, cell and cell group, cellular component, nucleic acid, organism, entity feature, life stage, phenotype, strain, sex, drugs and small molecules, molecular function, mutant, and clone. We have incorporated the GO molecular function category and proteins in the Textpresso molecular function category. A more detailed list with definitions can be found on the Textpresso Web site, and the most important ones are provided in Table 1. Many of these categories have subcategories. For example, the molecular function category has the subcategories “source = (Go|Textpresso)” and “protein = (yes|no).” As we have imported all terms from GO, the first subcategory makes it possible to search specifically for GO terms. Terms added by us have the attribute “Textpresso.” Similarly, not all molecular function terms are classified as protein. The word “co-transporter,” for example, conveys more of a function and would be used more in this context in the literature, even though its physical realization may in fact be a protein. A list of all subcategories can be found in Table 2.

**Table 1:**
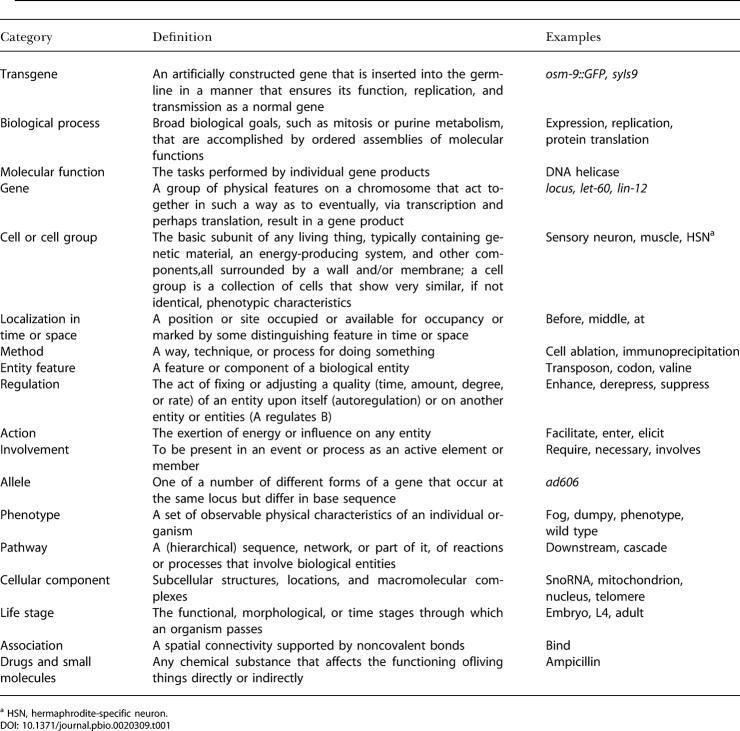
The 18 Biologically Most Relevant of the 33 Categories of the Textpresso Ontology

^a^ HSN, hermaphrodite-specific neuron

**Table 2:**
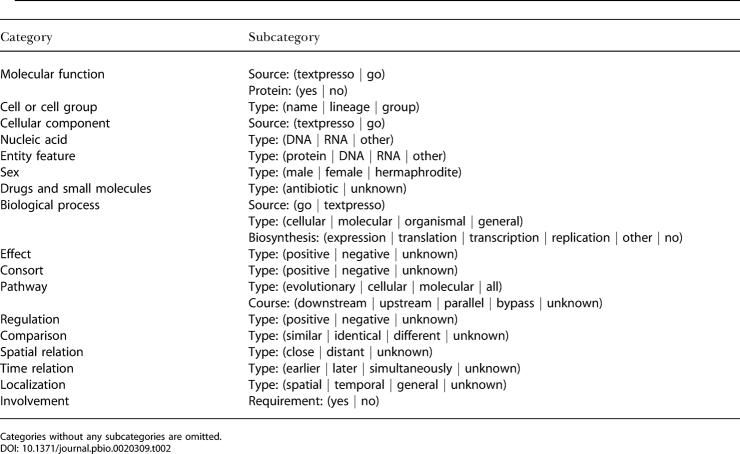
The Subcategories of the Ontology

Categories without any subcategories are omitted

The second group of categories comprises terms that characterize a biological entity or establish a relation between two of them. It includes physical association (in the sense of binding) and consort (abstract association), effect, purpose, pathway, regulation, comparison, spatial and time relation, localization in time and space, involvement, characterization (terms that express the characterization of something), method, biological process, action, and descriptor (words that describe the state or condition of an entity). These categories, while well defined, have somewhat delicate boundaries, and the common-sense aspects of our ontology apply more to this group. It is likely that its categories are going to be changed as we continue to develop the system. In some instances terms are attributed to one category, even though they might as well fit into another. As an example, the term “coexpress” is put in the “consort” category to emphasize the concurrent aspect of the process, while it could as well be classified as a biological process. However, we believe that in most cases the first sense of the word is used in the literature.

The last group (auxiliary) contains categories that can be used for more involved semantic analysis of sentences. These categories are auxiliary (forms of the verbs “be” and “have”), bracket, determiner, conjunction (and, or, because, since, although, etc.), conjecture (could, might, should, suggests), negation, pronoun, preposition, and punctuation. Some of them overlap with the syntactic categories that the part-of-speech tagger (used in the preprocessing steps; see Materials and Methods) assigns to terms, but are repeated here as they also contain some semantic component. The category “conjecture” is introduced to distinguish statements that convey hypotheses, speculations, or theoretical considerations from sentences that are expressed with confidence, thus representing more of a fact. The words of this category indicate the certainty of a statement.

The Textpresso ontology is organized into a shallow hierarchy with 33 parent categories. The parent categories may have one or more subcategories, which are specializations of the parent category. For example, all of the terms in the parent category “biological process” will belong to one of its subcategories, “transcription,” “translation,” “expression,” “replication,” “other,” or “no biosynthesis.” This is user friendly and certainly serves the current implementation of the user interface well, which is oriented more towards information retrieval.

**Figure 1:**
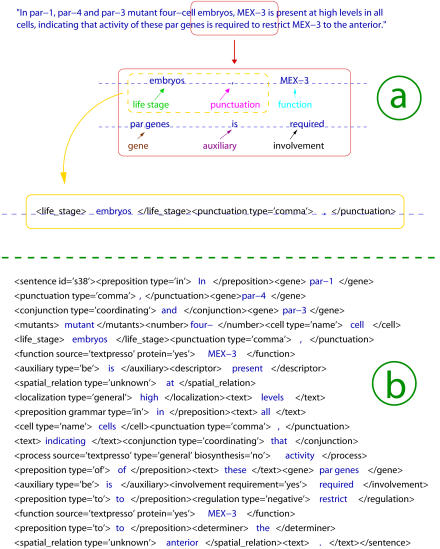
The Process of Marking up a Sentence The process of marking up the sentence “In par-1, par-4 and par-3 mutant four-cell embryos, MEX-3 is present at high levels in all cells, indicating that activity of these par genes is required to restrict MEX-3 to the anterior.” This sentence is taken from Huang et al. (2002). (A) The computer identifies terms that are stored in a lexicon according to categories of the ontology. A text-to-XML converter marks up the terms by enclosing them in XML brackets. (B) The fully marked-up sentence. Some categories have subcategories (for example, the category “regulation” is subdivided into “positive,” “negative,” and “unknown”). Grammar attributes have been omitted here for the sake of clarity, because they are not used in the current version of the system. Some white spaces have been inserted in the graphics for clarity enhancement.

The ontology is populated with 14,500 Practical Extraction and Report Language (PERL) regular expressions, each of which covers terms with a length from one to eight words. These expressions are contained in a lexicon. Table 3 shows examples of regular expressions for each category and examples of text strings matching them. Each regular expression can match multiple variable patterns. The multiple forms of regular verbs, for example, can be conveniently expressed as “[Ii]nteract(s|ed|ing)?” which stands for the eight cases “interact,” “interacts,” “interacted,” “interacting,” “Interact,” “Interacts,” “Interacted,” and “Interacting.” All regularly named C. elegans genes are matched with the expression “[A–Za–z][a–z][a–z]–\d+” matching three letters ([A–Za–z][a–z][a–z]), a dash (–), and a sequence of digits (\d+). As this example illustrates, the expressions can be made case sensitive. This is important as biological nomenclature becomes more elaborate, and the ability to distinguish subtle differences is pivotal for separating terms into the correct categories. Many of the regular expressions are generated automatically via scripts, taking a list of plain words as input and transforming them as shown in this example, to account for regular forms of verbs and nouns. The text-to-XML converter (see Materials and Methods) marks up the whole corpus of abstracts, full texts, and titles and produces XML documents. Figure 1 illustrates this process with an example. The computer identifies terms by matching them against regular expressions (such as the one shown above) and encloses them with XML tags. The tag <text> serves as a containment of terms not semantically marked up. These tags will be used for a repeated reevaluation of the lexicon, as these terms can be easily pulled out and analyzed. A list of the most frequently missed terms is then produced and included in the lexicon for the next markup.

**Table 3:**
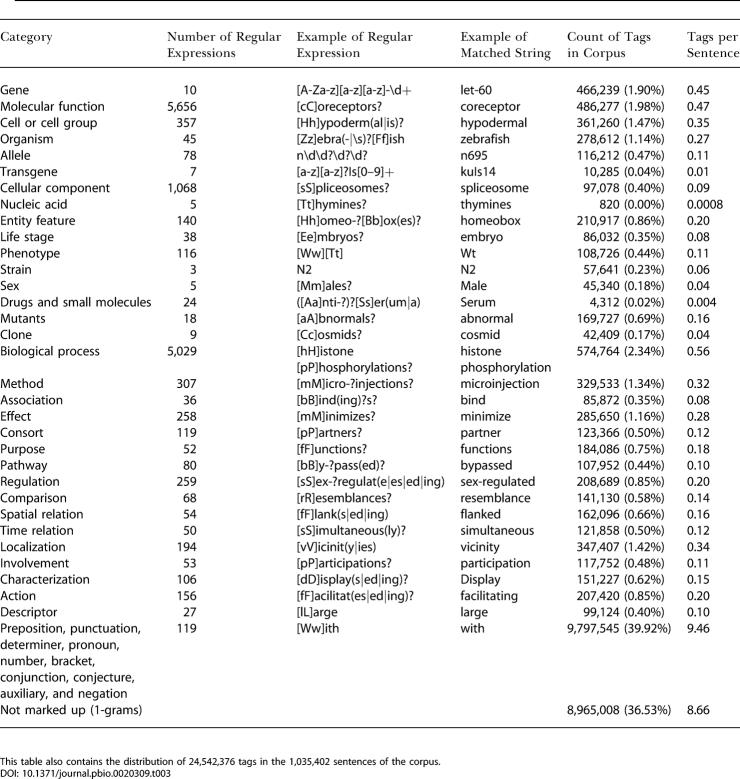
Categories of the Ontology with Examples of Regular Expressions and Matching Text Strings

This table also contains the distribution of 24,542,376 tags in the 1,035,402 sentences of the corpus

### Applications of Textpresso

The marked-up text is stored in a database and can be queried. We built a user interface for general queries and another one for a specific type of query for WormBase curators (gene-gene interactions; see below). Textpresso is used in several related ways. Individual biologists use it to find specific information. Database curators, whose job is to extract information from papers or abstracts and to add this to a database, use it repeatedly to find all information of a particular type, in addition to using it for individual queries.

**Figure 2:**
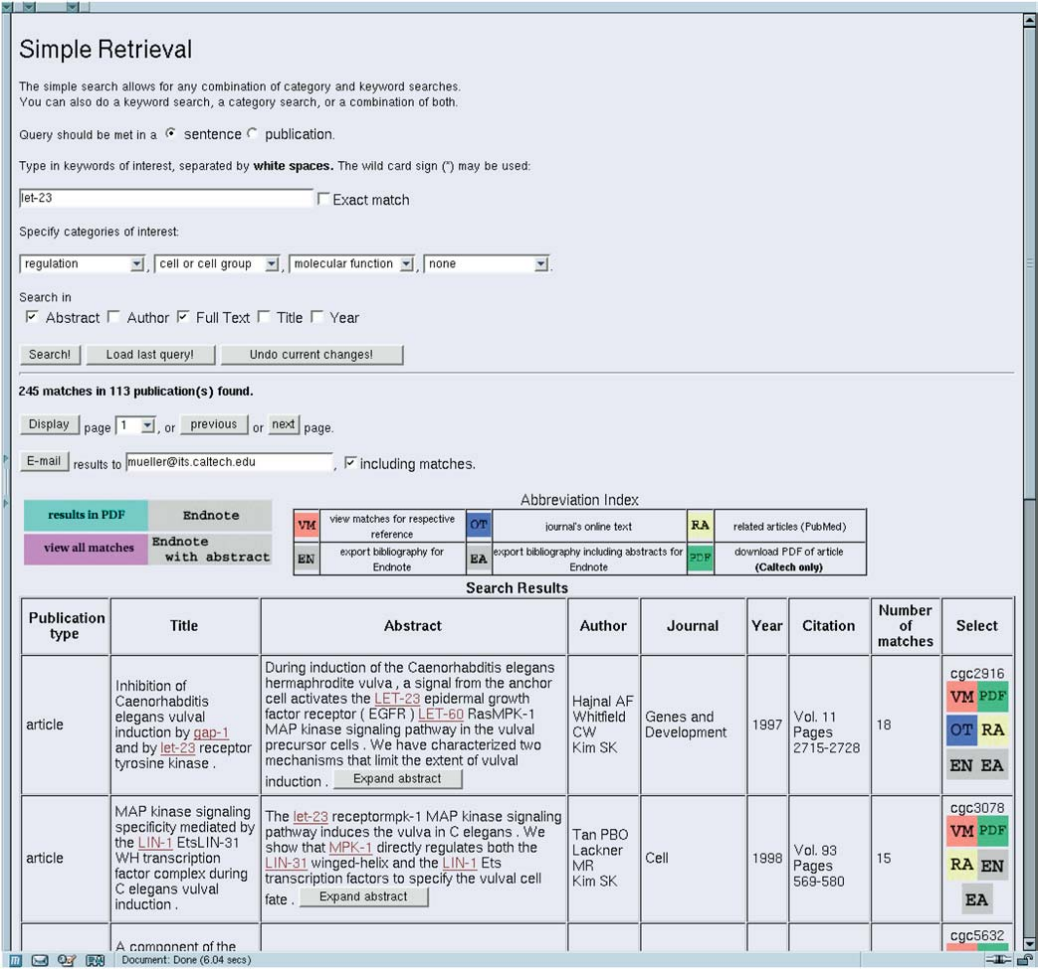
A Typical Result Page Returned from a Simple Retrieval Query (Keyword) A simple retrieval was performed with “let-23” as keyword and “regulation,” “cell or cell group,” and “molecular function” as categories. A total of 245 matches were found in 113 publications.

The current Textpresso user interface (http://www.textpresso.org/) includes a query interface, a side menu with links to informative pages about the ontology, a document type definition, a user guide, and example searches, as well as the two retrieval and customization interfaces. The Web site offers two different types of retrieval, simple and advanced. Options for the retrieval queries are offered: searching a combination of categories, subcategories, and keywords in a Boolean fashion, specifying the frequency of occurrences of particular items, and choosing where in the article to search (title, abstract, body). The user can also determine whether a query is to be met in the whole publication or in a sentence. These options make the search engine powerful; for example, if a query is met in the whole article, the search has the function of text categorization, while meeting it in a sentence aims at extracting facts, which can be viewed in the context of a paragraph. The specification of cooccurrence determines the character of a search. If a combination of keywords and categories is found in a sentence, the likelihood that a sentence contains a fact involving the chosen categories and keywords is quite high. If the user chooses cooccurrence within a document, he is more interested in finding a relevant document. The scope of a search can be confined to full text, abstract, title, author, year, or any combination thereof, for document searches as well as sentence searches. A typical result page shows a list of documents with all bibliographical information and the abstract as displayed in Figure 2. A simplified version of the Textpresso interface is incorporated within WormBase (http://www.wormbase.org).

The result list retrieved by a query can be customized in such a way that the user can choose how to display the information. This list is sorted according to the number of occurrences of matches in the document, so the most relevant document will be on the top of the list. A series of buttons for the whole list as well as for each document is available, allowing the user to view matching sentences or prepare search results in various formats. The individual result entries have up to six links: One can view matches for each paper only, go to the Web site of the journal to read the online text of the article (this only works if the user is subscribed to the journal), view a list of related articles that is provided by PubMed, export the bibliographical information into Endnote (two different links), or, if the user is accessing Textpresso internally (currently at Caltech), one can download the PDF of the paper.

The power of Textpresso's search engine unfolds when category searches are used. By searching for a category, the researcher is targeting all keywords that populate that category. For example, the researcher might be interested in facts about genetic regulation of cells. Assuming that many facts are expressed in one sentence, he would search for the categories “gene,” “regulation,” and “cell or cell group” in a sentence. He can then view the matches (and surrounding sentences) of the search return and decide which facts are relevant. If one is not interested in all genetic regulation instances mentioned in the literature, it might be more useful to combine keywords with categories. For example, the question “What entities interact with ‘daf-16' (a C. elegans gerontogene)?” can be answered by typing in the keyword “daf-16” and choosing the category “association.”

### Advanced Retrieval and Subcategories

An extension (the advanced retrieval interface) allows the use of the subcategories of the ontology and the specification of Boolean operators, thereby concatenating categories and keywords with “or” or “not” to permit alternatives or exclude certain items. One special subdivision of terms is the distinction between named and unnamed entities: Categories can include both general terms and specific names of entities. For example, the word “gene” would be an unnamed term of the gene category, while “lin-11” is a named entity. The general terms will likely be used for fact extraction across several neighboring sentences, but they might also be useful for retrieval purposes, even though the rate of false positives might be much higher in the latter case. Lastly, the user can determine how a keyword or category term has to be matched numerically. The options “greater than,” “less than,” and “equal to” are available together with a drop-down menu for the number of occurrences.

With these additional tools, document categorization can be made more effective. A detailed profile of which categories and keywords should occur a minimum, maximum, or exact number of times for triggering a match can be established. Similarly, searches on the sentence level acquire a semantic quality, i.e., they at least partially encompass a meaning. In many cases, the answers to questions, phrased in the form of a sophisticated query, can immediately be read off the result screen. If, for example, one were to ask in which cells *lin-11* is expressed, one would search sentences for a combination of the category “biological process” (subcategory “biosynthesis: expression”), the category “cell or cell group” (subcategory “type: name”) and the exact keyword “lin-11.” The subcategory “expression” filters out all words that relate to expression, the subcategory “name” limits the search to specific cells which have a name, such as “anchor cell,” “HO neurons,” “IL sensillum,” etc. Other subcategory options would be “group” (for example, “head,” “vulva,” “tail”) and “lineage” (“AB lineage,” “EMS lineage,” etc.). To better understand the following results, note that the term “cell(s)” has the type “name,” to gain the correct meaning of phrases such as “AB lineage cells.” The first two words of this phrase are marked as lineage, but the last word makes the whole phrase named cells.

The system returns sentences of different quality. Some of them answer the question posed immediately (returned sentences are taken from Gupta and Sternberg 2002; that paper produced the most hits). The underlined words mark the matched items: “An analysis of the expression pattern of lin-11 in *vulva and uterine lineage* cells earlier suggested that cellular defects arise due to a failure in the differentiation process”; “Our analysis of the expression of lin-11 in VPC granddaughters (Pn.pxx stage) has revealed the following pattern in *P5.p and P7.p lineage* cells (from anterior to posterior; L, low; H, high), LLHH and HHLL , respectively.” Other sentences meet the truth more by accident, as the terms are matched within a sentence, but the statement does not really express the fact sought. The cells where *lin-11* is expressed might be inferred by the knowledgeable reader, and not stated explicitly: “Our results demonstrate that the tissue-specific expression of lin-11 is controlled by two distinct regulatory elements that function as independent modules and together specify a wild-type egg-laying system”; “Using a temporally controlled overexpression system, we show that lin-11 is initially required in vulval cells for establishing the correct invagination pattern.” Finally, some sentences just do not give any clue about the posed question: “lin-11 cDNA-expressing vectors under the control of lin-11-AB (pYK452F7-3) and lin-11-C (pYK452F7-2) elements were designed as follows.” Here, “AB” is marked up as a named cell, but this is not the semantically correct tag in this context. This false positive might have been prevented if specific sections of a paper could be searched, as this statement comes from the method section.

### Evaluation of the Textpresso System

An automatic method for retrieving or extracting information from text is only useful if it is as accurate and reliable as human curation. We devised two tests based on two common tasks performed by human experts who extract biological data from journal articles. The first task was the automatic categorization of papers according to the types of biological data they contain. Our study used a large test set of papers scanned by a curator to examine the effectiveness of automatically searching for information in the full text of a journal article compared to its abstract. The second task focused on retrieving sentences containing a specific type of biological data from text. Sentences from eight journal articles were manually inspected on a sentence-by-sentence basis and compared to the return from a Textpresso query on the same articles. From this study we present a detailed error analysis outlining the strengths and weaknesses of the current Textpresso system as an automatic method for information retrieval.

We evaluated the performance of Textpresso using the information extraction performance metrics of precision, which is a measure of the amount of true returned data compared to the amount of false returned data, and recall, which is a measure of the true data returned compared to the total amount of true data in the corpus. These values are formulated as *recall* = *number of true returns* / *total number of true data items* and *precision* = *number of true returns* / *total number of returns.*

### Classification of Journal Articles: Full Text Versus Abstract

We examined the effectiveness of automatically identifying journal articles that contain particular types of data. A test set of 965 journal articles pertaining to C. elegans biology was assessed by a human expert and categorized into groups according to six different types of data (antibody data, ablation data, expression data, mapping data, RNAi data, and transgenes). Note that there can be more than one data type per article.

We first measured the value of searching for keywords in the full text of an article as opposed to searching its abstracts (Table 4). The overall information recall when searching abstracts is low (∼44.6%) compared to the information recall when searching full text (∼94.7%). Furthermore, keywords for some specific types of data (e.g., antibody data, mapping data, transgene data) are very unlikely to appear in abstracts (∼10% recall) but can be found in full text (∼70% recall). However, precision of the keyword search is reduced by almost 40% when searching full text compared to abstracts (30.4% and 52.3%, respectively). Single keyword searches of full text return a large number of irrelevant documents for most searches. This higher false positive rate might reflect the writing style found in full text, where facts can be expressed within complex sentence structures (as compared to abstracts, where authors are forced to compress information), combined with the inability of a keyword search to capture context.

**Table 4:**
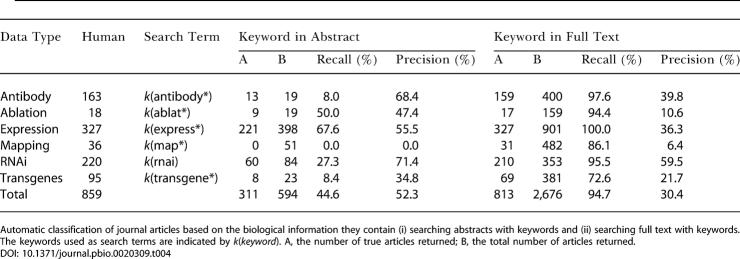
Comparison of a Keyword Search on Abstracts versus Full Text

Automatic classification of journal articles based on the biological information they contain (i) searching abstracts with keywords and (ii) searching full text with keywords. The keywords used as search terms are indicated by *k*(*keyword*). A, the number of true articles returned; B, the total number of articles returned

### Small-Scale Information Retrieval Study

**Figure 3:**
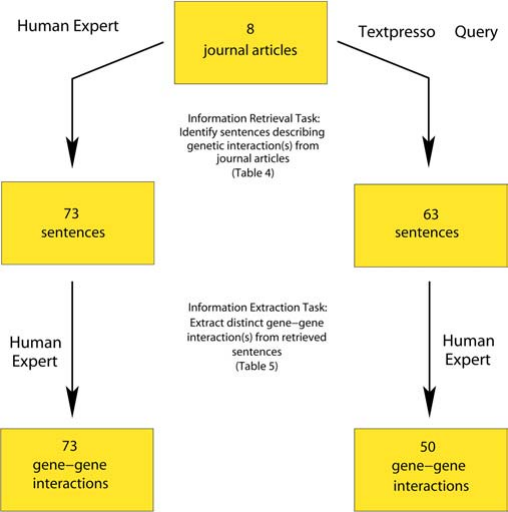
Schema of Small-Scale Information Retrieval Study Sentences from eight journal articles were both queried by Textpresso and evaluated by a human expert for sentences that described genetic interaction (information retrieval task). In the information extraction task, a human expert inspected the sentences returned by each method to determine the amount of distinct gene-gene interactions that could be extracted in order to analyze the output of the first task.

We tested the accuracy of a search combining word categories and keywords to retrieve sentences containing genetic interaction data. For this experiment we broadly defined genetic interaction as the effect of one or more genes on the function of another gene or genes (and thus it includes genetic interaction, regulation, and interaction of gene products). To directly assess how Textpresso performs, a human expert manually evaluated the text sentence by sentence (Figure 3).

We formulated a Textpresso query that searched for the presence of at least two genes mentioned by name and at least one term belonging to the “regulation” or “association” word categories (see Materials and Methods). A total of 178 sentences were matched for this query in the eight journal articles, and the results are shown in Table 5. A human expert assessed the returned sentences and determined that 63 sentences contained gene-gene interaction data according to our criterion. The same set of journal articles had been independently manually evaluated for their description of genetic interactions, and 73 true sentences were identified. In both cases, information from the article title, abstract, contents of tables, and reference section was excluded. Sentences that described genetic interaction using the gene product name rather than the gene were also excluded from this study. To measure recall, we first determined the total number of sentences that contained genetic interaction data.

**Table 5:**
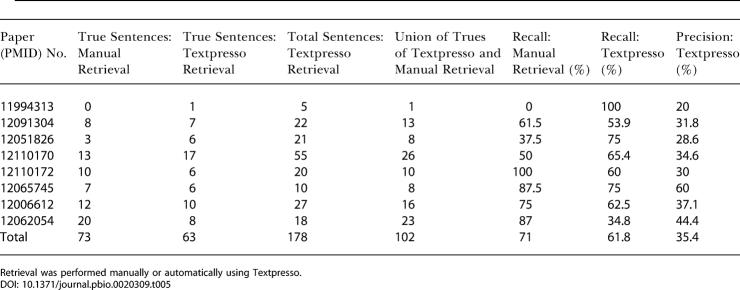
Retrieval of Sentences Containing Gene-Gene Interaction Data from a Set of Journal Articles

Retrieval was performed manually or automatically using Textpresso

For this analysis we took the union of true sentences manually identified in the journal articles and the true sentences returned by Textpresso. The total number of true sentences identified by the two methods was 102. The recall of sentences containing genetic interaction was ∼62% using Textpresso compared to ∼71% for those sentences manually identified in journal articles. One-third of the sentences returned by Textpresso were true positives (35%).

Although the numbers of true sentences retrieved by the automatic and manual methods were similar (63 and 73, respectively), only 34 of these sentences overlapped. To investigate this discrepancy, we manually extracted the genetic interactions described in both sets of sentences and determined the number of distinct genetic interactions found by each method (Table 6). The sentences manually identified from the journal articles yielded 23 more distinct genetic interactions than those which were extracted from true sentences retrieved by Textpresso. However, 43 interactions derived from the Textpresso output overlapped with the manually identified set, and Textpresso located sentences describing seven genetic interactions that the human expert missed. The average redundancy (how many times the same gene-gene interaction occurred) of a distinct genetic interaction extracted from both the manual and automatic methods was 3-fold.

**Table 6:**
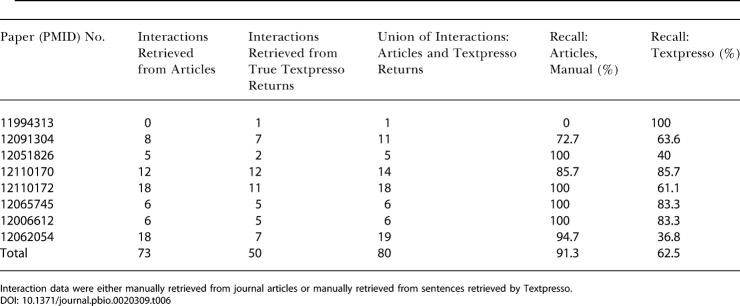
Distinct Gene-Gene Interactions Retrieved from Journal Articles

Interaction data were either manually retrieved from journal articles or manually retrieved from sentences retrieved by Textpresso

We analyzed the gene-gene interaction sentences missed by Textpresso. In many cases (65%) the word or phrase used to describe the genetic interaction belonged to neither the “association” nor the “regulation” word category and so the sentence was not returned. In some cases, the term or phrase that determined “genetic interaction” belonged to some other Textpresso word category (e.g., some terms that implied genetic interaction and were not matched by the query were “epistatic,” which belongs to the “consort” word category, and “alters,” which belongs to the “effect” word category). This type of analysis is useful for revising and updating the ontology. In other cases, due to the intricacies of natural language prose, it was difficult to isolate an interaction term in the sentence (e.g., “Thus *ref-2* alone is insufficient to keep P(3–6).p unfused when *lin-39* is absent.”). Approximately 8% of true sentences were missed because the genetic interaction information was discussed over a number of sentences. This is a limitation of the current Textpresso system, as search queries are matched per sentence (or per entire article).

Our analysis of the false positive sentences returned by Textpresso revealed that approximately 10% discussed gene-gene interactions that did not occur (e.g., “Neither *pdk-1(gf)* nor *akt-1(gf)* suppressed the Hyp phenotype of *age-1(mg44)*.”). While we do have a “negation” category in our Textpresso ontology, we chose not to exclude negation terms from the posed query, to avoid missing true positives (in case the negation does not apply to the interaction term in a sentence, but to some other portion of it). Twenty-one percent of the false positive sentences were determined by inspection to suggest genetic interaction, but were too weakly phrased to extract the information in confidence without the context of the sentence. However, the majority of false positives (70%) were due to the lack of context of the search terms in the sentence, where they matched the query terms (underlined) but in a context that did not mention genetic interaction: “lin-35 and lin-53, two genes that antagonize a C. elegans pathway, encode proteins similar to Rb and its binding protein RbAp48.” This example strongly supports the idea that an information extraction method that considers semantic context of a search query would dramatically increase the precision of the return.

### Large-Scale Information Retrieval to Expedite Information Extraction

We performed extraction of genetic interaction information from a corpus of 3,307 journal articles. A Textpresso query searched for the presence of at least two uniquely named genes and at least one term belonging to the “regulation” or “association” word categories (see Materials and Methods for more details). A total of 17,851 sentences were returned by this query. Due to the lack of context of some sentences, true sentences were determined by a more stringent definition of genetic interaction, i.e., where one or more named genes were described as modifying the phenotype of another named gene or genes by suppression, enhancement, epistasis, or some other genetic method. To determine the frequency of true sentences, a random sample of 200 of the sentences returned by Textpresso was evaluated by a human expert according to this more stringent criterion (Table 7, column C). This sample was compared to 200 sentences chosen from the whole corpus at random (Table 7, column A) and 200 sentences randomly chosen from the whole corpus that contained two or more named genes (Table 7, column B).

**Table 7:**
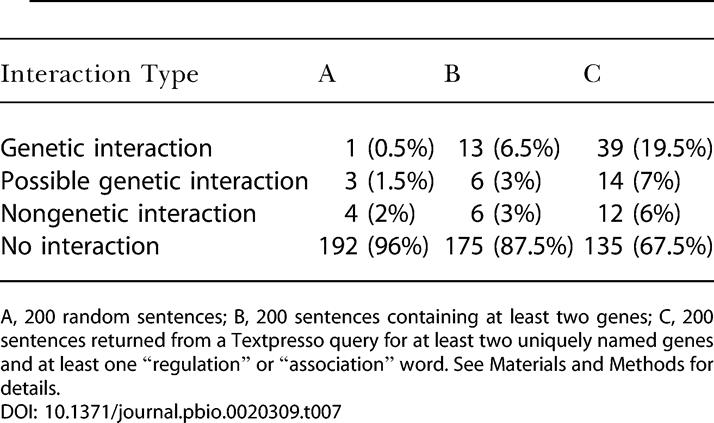
The Frequency of Genetic Interaction Data Contained in Full Text

A, 200 random sentences; B, 200 sentences containing at least two genes; C, 200 sentences returned from a Textpresso query for at least two uniquely named genes and at least one “regulation” or “association” word. See Materials and Methods for details

A typical sentence that was determined to be true for genetic interaction data is “Interestingly, at lower temperatures, the *akt-2(+)* transgene can supply sufficient Akt/PKB activity to weakly suppress the dauer arrest caused by *age-1(mg44)*.” Some of the sentences strongly suggested genetic interaction but did not quite meet the genetic interaction criterion. These were grouped as “possible genetic interaction,” for example, if a phenotype was not mentioned: “For example, *lin-15(lf)* animals display a 54% penetrance of P11 to P12 fate transformation, while all *egl-5(lf)*;*lin-15(lf)* double mutants show a P12 to P11 fate transformation.” Sometimes it is unclear exactly which genes are participating in the genetic interaction: “Evidently the effect of the sir-2.1 transgene alone is too subtle to trigger dauer formation without the sensitizing *daf-1* or *daf-4* mutations.” Another group was highlighted as discussing interaction, but fell outside the criterion set for genetic interaction. These were classified “non-genetic interaction.” Some examples of this are sentences that specify gene regulation: “These studies have shown that *smg-3(Upf2)* and *smg-4(Upf3)* are required for SMG-2 to become phosphorylated.” Finally, sentences that describe physical interaction were also put into the category “possible genetic interaction”: “For example, GLD-1 represses translation of *tra-2*, one of the sex-determination genes, by binding to the 3′-UTR or the *tra-2* mRNA (Jan et al. 1999).”

This analysis shows that there is a 1 in 200 chance of a sentence discussing genetic interaction (as defined above) randomly occurring in the full text of the journal articles analyzed. The odds increase to 7 in 100 if one looks at sentences containing at least two named genes. The returned matches from the Textpresso search are enriched 39-fold for genetic interaction compared to random chance, and there is a significant 3-fold enrichment when compared to sentences containing at least two named genes. There is a 1 in 5 chance that a returned Textpresso match is true. To date, 2,015 of the 17,851 returned sentences have been evaluated. Of these, 370 discuss genetic interaction, yielding 160 distinct gene-gene interactions mined from the literature. There are 213 sentences that mention nongenetic interactions, and 419 sentences are classified as possible genetic interactions.

### Large-Scale Simple Fact Extraction

We have extracted gene-allele reference associations from the corpus of papers to populate the WormBase database by searching for the pattern <gene><bracket><allele> <bracket>. Of the 10,286 gene-allele associations extracted, 9,230 were already known by WormBase, while 1,056 associations were new and could be added to the database. In addition, 1,464 references could be added to the 2,504 allele reference associations in WormBase. Ninety-eight percent of the data extracted went into the database without any manual correction, and the last 2% were compromised because of typographical errors in the original paper or the inherent character of the data (i.e., gene name synonyms and changes).

## Discussion

### Accomplishments

We have developed a system to retrieve information from the full text of biological papers and applied it to the C. elegans literature. As of March 2004, the database contains full texts of 60% of all papers listed by the *Caenorhabditis* Genetics Center (CGC; http://www.cbs.umn.edu/CGC/CGChomepage.htm) and almost all abstracts that are information rich for C. elegans research. The introduction of semantic categories and subsequent marking up of the corpus of texts introduce powerful new ways of querying the literature, leading towards the formulation of meaningful questions that can be answered by the computer. We have demonstrated such queries with one example and have successfully tried many others. A more thorough evaluation of the system revealed that the availability of full text is crucial for building a retrieval system that covers many biological data types with a satisfying recall rate, and thus is truly useful for curators and researchers. For biologists, an automated system with high recall and even moderate precision (like the current Textpresso) confers a great advantage over skimming text by eye. Textpresso is already a useful system, and thus serves not only as proof of principle for ontology-based, full-text information retrieval, but also as motivation for further development of this and related systems to achieve higher precision and hence even greater time savings.

It is apparent that the number of articles available in the C. elegans literature (currently about 6,000) can be curated with the assistance of Textpresso, as it is much more efficient than when done by human readers alone. The larger the corpus of papers, the more useful Textpresso will become. We have shown this by calculating the frequencies of genetic interaction data in sentences in three different cases: random sentences, sentences that contain at least two genes, and sentences returned from a Textpresso advanced query. The efficiency was shown to increase dramatically (39-fold in the best case). We have outlined the first steps of how Textpresso helps the curation effort by extracting gene-gene interactions. Overall, we have shown that Textpresso has several uses for researchers and curators: It helps to identify relevant papers and facts and focuses information retrieval efforts. Indeed, Textpresso is used daily by C. elegans researchers and WormBase curators: The server sends 530 files to requests daily via the Web, a quarter of which are to WormBase curators.

### Areas for Improvement

Textpresso is limited in two ways: the lack of complete coverage of the C. elegans literature and the fact that the ontology and its corresponding lexicon are still in their infancy. The preparation of full texts has to be better and more efficient. The conversion of PDF to plain texts was problematic because of the different layouts of each journal. Even with the software we developed, a layout template for each journal needs to be written to specify where different components of text can be found. Prior to the use of this software, we had to forgo the use of figure and table captions. Acquisition of processable text is a general problem for biologists. A new release of XPDF (a PDF viewer for X; http://www.foolabs.com/xpdf/) eases this problem considerably (see Materials and Methods).

One of our studies on the effectiveness of the extraction of a specific type of biological fact, in this case gene-gene interaction, showed that the machine still cannot replace the human expert, although it increases efficiency greatly. We anticipate that the computer does better with a larger number of articles because of redundancy. While roughly 9% of distinct gene-gene interactions from a corpus of eight journal articles were missed by the human but revealed by Textpresso, 29% of the interactions were missed by Textpresso, primarily due to flaws in the ontology.

Advancing the Textpresso ontology will help to increase the specificity of the retrieval system. A deeper, meaningful structure is likely to make extraction easier and more stable. Possible improvements are to include other biological ontologies and language systems, such as UMLS (http://www.nlm.nih.gov/research/umls/) and SNOMED (http://www.snomed.org/, and to establish a more sophisticated tree structure. Our core lexicon recognizes 5.5 tags per sentence (out of an average of 23.7 tags per sentence) that are of scientific interest. This density results in a term coverage of 23.2%, while the maximum that could theoretically be added is 36.5%, assuming that all terms currently not marked up belong to relevant categories. An average of 9.5 tags per sentence are apparently of no interest for information retrieval; however, this is due to the nature of human language (and will be nonetheless useful for information extraction purposes). Reevaluation of the corpus of text for terms and their meanings that have been missed is necessary. This process will result in an expansion of our ontology, thus continually expanding the resulting lexicon, or revising the structure of the ontology. Ontology and lexicon revision is most efficiently done by a human, and a feasible automated approach seems out of reach. However, we have illustrated semiautomatic methods to help make this task easier in the future: The containment of words that are not covered in our lexicon with <text> tags serves several purposes. First, we are able to extract all words (or n-grams, which are represented as a consecutive sequence of words embedded in <text> tags), assemble a histogram of the most frequent terms, and add important ones to our lexicon. Second, having identified frequently occurring semantic patterns in the corpus, we are able to infer likely candidates of words for specific categories. For example, one popular pattern that indicates a gene-allele association is <gene><bracket><allele><bracket>. If one now searches for patterns such as <gene><bracket> <text><bracket> and extracts the word enveloped by the <text> tags, then a frequency-sorted list of words that are likely to be alleles can be assembled, presented to a curator for approval, and deposited into the lexicon. The alternative, <text><bracket><allele><bracket>, would give a list of possible gene names. Many other patterns, identified by statistical means and similarity measures, could be obtained and used in such a fashion. These two methods will help us to systematically and significantly reduce the number of terms not marked up in the corpus, making it more complete. The procedure can be repeated with every build of the Textpresso database and has the advantage that the list of words added to the lexicon is tailored to the literature for which it is used. In addition, shortcomings in the general structure of the ontology can be detected and corrected, if those issues have not been caught in the research and development of the information extraction aspects of the system. If the strategy outlined above is applied continually, we will be able to close this gap and reach saturation, even with the addition of new papers and abstracts.

About 89% of current users take advantage primarily of the full text and multiple keywords. Some (11%) proceed to keyword plus category. Only 0.3% of users use the advanced retrieval search. It is clear that the implementation of a user test interface improvement/education cycle will greatly help the development of Textpresso and subsequently help users take full advantage of this system. More generally, biologists will become increasingly familiar with ontology-based search engines.

### Prospects

Future development of Textpresso can be undertaken at many different levels. A synonym search could be enabled for keyword searches: After having compiled lists of them, an option could be given to automatically include synonyms for a given term (e.g., genes, cells, cellular component) in a search. Similarly, GO annotations could be used to search for and display sentences involving genes associated with gene ontology terms, after the latter ones have been queried first. As already mentioned, search targeting could be made more flexible: Papers could be subdivided into more sections (such as introduction, methods, results, conclusion, etc.), and a query could then be applied only to the specified sections. In addition, the limitation of searching criteria to just one sentence can be relaxed to a set number of neighboring sentences. Finally, one could improve on links to other databases of relevance besides WormBase and PubMed and increase the wealth of links to the latter ones.

An important issue is the portability of the system to other model organism databases. This undertaking is part of the Generic Model Organism Database (GMOD) project (http://www.gmod.org, and a downloadable package with software will be made available on their Web site. For a different model organism, parts of the lexicon, and maybe also parts of the ontology, need to be modified. Language and jargon in each community differ, and terms need to be systematically collected to accommodate their specific usage in the respective communities. However, this is not too laborious, as we have been able to generate a yeast version in a few weeks (E. E. Kenny, Q. Dong, R. S. Nash, and J. M. Cherry, unpublished data).

We believe that Textpresso can be extended to achieve information extraction. The wealth of information buried in semantic tag sequences of 1 million sentences asks to be massively exploited by pattern-matching, statistical, and machine learning algorithms. Having the whole corpus semantically marked up provides bioinformaticians with the opportunity to develop fact extraction algorithms that might be quite similar to sequence alignment and gene-finding methods, or, more generally, algorithms that have similarity measures at their core, because sentences can now be represented as sequences of semantic tags. Furthermore, semantic sequences of related sentences show similar properties as related genomic sequences, such as recurring motifs, insertions, and deletions. The relatively rigid structure of the English language (subject-verb-object) and the comparatively low degree of inflections and transformations certainly help. In addition, some scientific information is stored in a structured manner. We have already started to run simple pattern-matching scripts to populate gene-allele associations from the literature for WormBase, as many of them are written in the form “gene name(allele name),” such as “lin-3(n1058).”

## Materials and Methods

### 

#### Sources.

**Figure 4:**
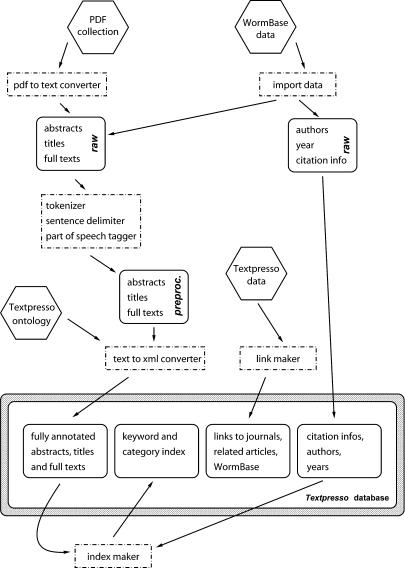
Schema of Textpresso Database Preparation The regular hexagons indicate the sources from which Textpresso is built. The rounded rectangles are either intermediate or final processed parts of the corpus. The dashed-dotted rectangles signify automatic processing units or actions.

Textpresso builds its C. elegans database from four sources. A collection of articles in PDF format is compiled according to the canonical C. elegans bibliography maintained at the CGC (http://www.cbs.umn.edu/CGC/CGChomepage.htm ). As of March 2004 we had around 3,800 (60%) CGC papers in our database. Software developed by us (see below) converts the PDFs to plain text. We import additional bibliographical information from WormBase: titles of documents and author and citation information. WormBase data comprise additional *C. elegans-*related documents such as C. elegans meeting abstracts and Worm Breeder's Gazette articles. We also curate certain types of data ourselves. Some *C. elegans-*related papers are not found in the CGC bibliography or WormBase. We compile lists of URLs of journal Web sites and their articles, and links to related articles (provided by PubMed). Citations are prepared in Endnote format for download. Finally, as Textpresso returns scientific text to the user, we construct links to report pages of WormBase that display detailed information about biological entities, such as genes, cells, phenotypes, clones, and proteins. All data and links produced by us are referred as “Textpresso” data in Figure 4.

#### Ontology.

The objective of an ontology is to make the concepts of a domain and the relationships and constraints between these concepts computable. For an ontology to be utilized in a search engine for biological literature, it has to include the language of everyday use and common sense. We have therefore assigned the most commonly used meaning to a word even though it has several meanings in different contexts. We have consequently adopted a strategy of devising an ontology drawing from our own knowledge. Our ontology includes all terms of the three major ontologies of GO, namely “cellular component,” “biological process,” and “molecular function.” The current ontology is unstructured for the sake of straightforward usability, our first priority.

A variety of approaches were utilized to construct and populate the 33 categories of the Textpresso ontology. We first designed individual categories for well-defined biological units or concepts such as strain, phenotype, clone, or gene. The terms in some of these categories (such as clone, allele, and gene) were represented by a PERL regular expression designed to match any text that looked like that particular biological unit. This was possible where a conserved and unique nomenclature for that biological concept had been established in the literature. Any exceptions to the established nomenclature recorded in WormBase were also added to these categories.

For other biological concepts (e.g., “method,” “phenotype,” “cellular component,” and “drugs and small molecules”), we extracted information from publicly accessible biological databases, such as WormBase, GO, and PubMed/NCBI to construct lists of terms. We supplemented these lists through primary literature and textbook surveys.

Next, we conceived categories of terms that would describe the relationship between the biological categories. To structure these “relationship” categories, we listed words of the text of 400 C. elegans journal articles for analysis. From this list we flagged natural prose words that we felt had at least some defined meaning within the context of biological literature (for example, “expressed,” “lineage,” “bound,” “required for”). From this list we constructed 14 new categories designed to encapsulate the natural language used by biologists to describe biological events and the relationship between them (action, characterization, comparison, consort, descriptor, effect, involvement, localization in time and space, pathway, purpose, physical association, regulation, spatial relation, and time relation). We made a second pass through the subset of flagged words from the list and assigned them to one of these categories according to what the sense of the word was in the biological literature for the majority of the time.

Finally, a number of categories were designed to account for syntax and grammatical construction of text, such as “preposition,” “conjunction,” and “bracket.”

#### Names.

We have manually curated a lexicon of names because it has proved difficult in the past to automatically recognize names of biologically relevant entities (Fukuda et al. 1998; Proux et al. 1998; Rindflesch et al. 2000; Blaschke and Valencia 2002; Hanisch et al. 2003). We therefore chose to curate and maintain a lexicon with names of interest by hand. In this *C. elegans-*specific implementation of Textpresso, the effort was helped by the fact that the C. elegans community is somewhat disciplined in choosing names and WormBase includes names of interest. Of course, there is the danger that entities not listed in WormBase (and therefore in our lexicon) will be missed in our system, and those cases are of special interest to curators (of WormBase) and researchers, such as newly defined genes or newly isolated alleles. Dictionaries tend to be incomplete and turn stale rapidly, because of the issues of synonyms, lack of naming conventions, and the rapid pace of scientific discovery. Thus, we do not rely only on WormBase, but maintain an independent, Textpresso-specific part of the lexicon.

#### Technical aspects of the system.

Figure 4 shows the details of database preparation. The regular hexagons indicate the sources from which Textpresso is built. The PDF collection was converted to plain text by a software package written by Robert Li at Caltech. The development of such a software tool had become necessary, as current PDF-to-text converters do not comply with the typesetting of each journal, i.e., footnotes, headers, figure captions, and two-column texts in general are dispersed and mixed up senselessly in the converted text. The application works with templates that specify the structure and fonts used in a particular journal and uses this information to convert the articles correctly. A high-fidelity conversion is crucial for any information retrieval and extraction application. The software will be made available at the GMOD Web site (http://www.gmod.org). While this manuscript was being written, a new version (2.0.2) of XPDF (http://www.foolabs.com/xpdf/) was released. This version, unlike its predecessors, does a superb job in converting PDF into a congruent stream of plain text.

Additional bibliographic data of references for which PDFs are not available are imported from WormBase (symbolized as “WormBase data” in Figure 4). These are mainly abstracts from various meetings. The data collected from our primary sources are treated in two different ways. Author, year, and citation information are deposited “as is” into the database, while abstracts, titles, and full texts are further processed. First, the texts are tokenized. Our tokenizer script reads the ASCII text derived from the conversion from PDF and splits the text into individual sentences based on the end-of-sentence period, where words hyphenated at the end of a line are concatenated and instances of periods within sentences (which are used mainly in technical terms and entity names) are ignored. The script also adds an extra space preceding any instance of punctuation within a sentence, which is a requirement for the Brill tagger (Brill 1992), a publicly available part-of-speech tagger, to attach 36 different grammatical tags to each tokenized word. The tagger has been trained specifically to handle the C. elegans literature, and additional tagging rules are applied. For example, gene names are forced to be tagged as nouns. The grammatical tags are not further used in the current Textpresso system. After this preprocessing step, the corpus of titles, abstracts, and full texts is marked up using the lexicon of the ontology (PERL expressions), as explained in Results and exemplified in Figure 1. The tags contain the name of the category as well as all attributes that apply to a matched term. Terms that are not matched by any of the 14,500 PERL expressions are given the tag <text>, one token at a time.

The corpus of searchable full texts, abstracts, and titles has 1,035,000 sentences. A total of 351,000 keywords have been indexed, covering 19,180,000 words in the texts. The semantic mark-up yields a total of 24,542,000 tags. Table 3 shows the distribution of tags. The number of meaningful tags (the ones that are not just <text>) is only 15,577,368, or 15.04 tags per sentence. An average of 5.5 tags per sentence are of scientific interest, i.e., are either biological entities or words that describe a relationship or characterize an entity.

When displaying sentences and paragraphs, Textpresso provides links to report pages of several biological entities, such as proteins, transgenes, alleles, cells, phenotypes, strains, clones, and loci. There are a total of 165,000 different entities in WormBase to which Textpresso links, including links to journal articles and PubMed. All these links are produced statically and again deposited on disk for fast retrieval, and these data are referred to as “Textpresso data” in Figure 4. In this way the actual link is not made on the fly from generic URLs, and the response time for queries remains short.

We generated an exhaustive keyword and category index for the whole corpus. This index makes the search extremely fast, using rapid file access algorithms. All keywords and tags in the corpus are indexed. Also, all terms in the corpus that have a report page in WormBase are indexed. For 2,700 full-text articles and 16,300 abstracts, the index takes up 1.7 Gb.

The interfaces for submitting queries and customizing display options are written as CGI scripts. They are supported by simple HTML pages that contain documentation. The Web site runs with a RedHat Linux operating system and an Apache http server. No special changes to the standard configuration are required. The Web interface accesses the custom-made Textpresso database; no commercial-grade database systems have been used. It takes 2–3 d to build the complete 6.9-Gb database.

#### Methodology of evaluation.

For the preliminary study, a query was formulated using three category rows of the Textpresso “advanced retrieval” interface to identify sentences containing gene-gene interaction data from a test set of eight full-text journal articles (see Table 5): the PMID:11994313 (Norman and Moerman 2002), PMID:12091304 (Alper and Kenyon 2002), PMID:12051826 (Maduzia et al. 2002), PMID:12110170 (Francis et al. 2002), PMID:12110172 (Bei et al. 2002), PMID:12065745 (Scott et al. 2002), PMID:12006612 (Piekny and Mains 2002), and PMID:12062054 (Boxem and van den Heuvel 2002). In the top row of the advanced retrieval tool the “association” ontology was selected in the “category or keyword” column. No other changes in the first row were made, which implies that no subcategory or specification was selected, and the occurrences of association terms in one sentence were “greater than 0.” In the second row, the Boolean operator “or” and the category “regulation” were selected, with no further specification, again asking the machine to return sentences with at least one regulation term. Finally, in the third row, the category “gene” was chosen, with a specification of “named” and an occurrence of “greater than 1.” The Boolean operator to connect this row with the former ones is “and.” All other values remained as default, resulting in no further query specification. As the “advanced retrieval” search engine processes queries sequentially from the top row to the bottom row, this query asks to return sentences with at least one association or regulation term in conjunction with at least two genes mentioned by name.

For the semiautomatic information extraction from text, the same query was utilized as above. In addition, sentences that did not mention at least two uniquely named genes were eliminated.

## Abbreviations

CGC: *Caenorhabditis* Genetics Center
GMOD: Generic Model Organism Database
GO: Gene Ontology
PERL: Practical Extraction and Report Language
PMID: PubMed unique identifier
SNOMED: Systemized Nomenclature of Medicine
UMLS: Unified Medical Language System
XML: eXtensible Markup Language
XPDF: a PDF viewer for X
